# Association of vitamin D deficiency with incident depression in patients with hearing impairment: an observational retrospective cohort study

**DOI:** 10.3389/fnut.2026.1856953

**Published:** 2026-06-10

**Authors:** Wan-Jung Cheng, Chia-Li Kao, I-Yin Hung

**Affiliations:** 1 Department of Anesthesiology, Chi Mei Hospital, Liouying, Tainan City, Taiwan; 2 Department of Anesthesiology, E-Da Hospital, I-Shou University, Kaohsiung City, Taiwan; 3 Department of Anesthesiology, Chi Mei Medical Center, Tainan City, Taiwan

**Keywords:** cohort study, depression, hearing impairment, propensity score matching, vitamin D deficiency

## Abstract

**Background:**

Hearing impairment is associated with an elevated risk of depression, yet modifiable risk factors in this population remain poorly characterized. Although vitamin D deficiency (VDD) has been linked to depression in general populations, no large-scale study has examined this association among adults with hearing impairment.

**Methods:**

This retrospective cohort study used the TriNetX Global Collaborative Network. Adults aged ≥18 years with hearing impairment who underwent serum 25-hydroxyvitamin D [25(OH)D] testing between 2010 and 2023 were classified into VDD (< 20 ng/ml) and sufficiency (≥30 ng/ml) cohorts. Thus, the study population represented a selected healthcare-utilizing cohort with available vitamin D measurements. After propensity score matching (48,184 per group), Cox models estimated hazard ratios (HRs) for incident depression over 12 years using a 1-year landmark design.

**Results:**

For the primary outcome, VDD was associated with a significantly higher risk of overall depression (*HR*, 1.57; 95% *CI*, 1.51–1.64; *P* < 0.001). For secondary outcomes, significant associations were observed for depressive episode (*HR*, 1.60), recurrent depression (*HR*, 1.62), suicidal behavior/self-harm (*HR*, 1.47), and all-cause mortality (*HR*, 1.40; all *P* < 0.001). The associations remained robust across sensitivity analyses (*HRs*, 1.55–1.65). Subgroup analyses showed consistent associations across sex, age, and comorbidity strata, with significant effect modification by age, obesity, and chronic pain. Vitamin D insufficiency (20.0–29.9 ng/ml) showed a smaller but significant association (*HR*, 1.37), consistent with an exposure-gradient pattern across predefined vitamin D categories. The negative control outcome (acute appendicitis) was non-significant (*P* = 0.267).

**Conclusions:**

VDD was associated with increased long-term depression risk among adults with hearing impairment who underwent serum 25(OH)D testing. Vitamin D status may represent a potential risk marker for depression in this selected healthcare-utilizing population; however, the observational design does not establish that vitamin D correction reduces depression risk.

## 1 Introduction

Depression is a leading cause of disability worldwide and is associated with substantial impairment in quality of life, daily functioning, and survival (1–4). Individuals with hearing impairment may be particularly vulnerable to depression, as hearing loss can hinder communication, increase social isolation, and adversely affect social relationships, independence, and participation in daily activities (5, 6). Communication difficulties may increase the effort required for daily social interaction, creating a persistent communication burden that can lead to frustration, avoidance of conversation, and reduced participation in social activities. Loneliness may further emerge when impaired hearing limits meaningful interpersonal exchange, particularly in older adults or individuals with progressive hearing loss. In addition, sensory deprivation may reduce environmental engagement and contribute to emotional distress, thereby providing a hearing-impairment-specific pathway through which depression risk may increase. These psychosocial consequences may accumulate over time and adversely affect emotional well-being. Given the high prevalence of hearing impairment (7–9), even a modest increase in depression risk may translate into a substantial clinical and public health burden. This association is supported by meta-analytic evidence showing that hearing impairment is linked to an approximately 1.3- to 1.7-fold higher risk of depression (10, 11). Therefore, identifying potentially modifiable risk factors for depression in this high-risk population is of considerable clinical importance.

Vitamin D deficiency (VDD) has been implicated as a potential risk factor for depression through several biologically plausible mechanisms, including impaired serotonergic neurotransmission, increased neuroinflammatory activity, and diminished neuroprotective effects (12–14). In the general population, observational studies and meta-analyses have consistently linked VDD to a higher risk of depression (15–17). Patients with hearing impairment may be particularly vulnerable to VDD because of reduced outdoor activity, social withdrawal, and poor nutritional status (18). However, these same factors may also be associated with depression risk and therefore may act as potential confounders rather than solely as explanatory pathways. Accordingly, evaluating the association between VDD and incident depression in patients with hearing impairment may help clarify whether vitamin D status is a relevant risk marker in this vulnerable population.

Despite this possibility, no large-scale longitudinal study has specifically examined whether VDD is associated with incident depression in adults with pre-existing hearing impairment. Clarifying this relationship may help determine whether vitamin D status is a clinically relevant risk marker in this vulnerable population. Therefore, we conducted a retrospective cohort study using the TriNetX Global Collaborative Network to investigate the association between VDD and the 12-year risk of incident depression in adults with hearing impairment.

## 2 Methods

### 2.1 Data sources

This study was conducted using the TriNetX Global Collaborative Network. The database contains demographic information, diagnoses coded according to the ICD-10-CM, procedural records, laboratory values, and medication orders. Analyses were performed using the TriNetX built-in analytics interface. The study protocol was reviewed and approved by the Institutional Review Board of Chi Mei Medical Center, which waived the requirement for informed consent owing to the use of de-identified data.

### 2.2 Exposure definition and index date

We identified adults aged ≥18 years with a documented diagnosis of hearing impairment (ICD-10-CM: H90, H91) who underwent serum 25(OH)D testing between January 1, 2010, and December 31, 2023. Patients were assigned to one of two cohorts according to their first qualifying 25(OH)D result: the VDD cohort, defined by a serum 25(OH)D level of < 20 ng/ml, and the control cohort, defined by a level of ≥30.00 ng/ml. Because TriNetX aggregates EHR data from multiple healthcare organizations, laboratory assay methods and calibration procedures were not standardized by the investigators across sites. Serum 25(OH)D values were analyzed as recorded in the source EHR systems. Information on season or month of vitamin D testing was not available in the analytic output and therefore could not be incorporated into exposure classification. The index date was set as the time of the first recorded 25(OH)D value meeting the respective cohort threshold, rather than the date of the first vitamin D measurement overall. To reduce exposure misclassification, a 5-year lookback period was applied. Patients in the VDD cohort were required to have no prior 25(OH)D values ≥30.00 ng/ml, whereas those in the sufficiency cohort were required to have no prior values < 20 ng/ml during this period. Specifically, 10,054 patients initially eligible for the VDD cohort were excluded because they had a prior 25(OH)D value ≥30 ng/ml, and 37,529 patients initially eligible for the sufficiency cohort were excluded because they had a prior 25(OH)D value < 20 ng/ml.

### 2.3 Exclusion criteria

To minimize reverse causation and ensure temporal separation between exposure and outcome, a 1-year landmark design was applied. Patients with depression-related conditions occurring before the index date or within 1 year after the index date were excluded. Specifically, patients with a depressive episode (F32), recurrent major depressive disorder (F33), suicidal ideation (R45.851), suicide attempt (T14.91), intentional self-harm (X71–X83), or osteoporosis with pathological fracture (M80) during this period were excluded. Osteoporosis with pathological fracture was excluded during the landmark period because it was prespecified as a positive control outcome; this exclusion ensured that positive control events were assessed as incident outcomes after the landmark period rather than as baseline or early post-index events. Additional exclusions applied before the index date included advanced chronic kidney disease (stages 4–5), end-stage renal disease or dialysis dependence, bipolar disorder, schizophrenia spectrum disorders, dementia of any type (including vascular dementia, Alzheimer's disease, and unspecified dementia), and major cerebrovascular or intracranial events (i.e., cerebral infarction, intracerebral hemorrhage, and intracranial injury; Supplementary Table 1). These exclusion criteria were intended to reduce confounding from conditions independently associated with both vitamin D status and the subsequent risk of depression. Follow-up for incident depression began 365 days after the index date and continued thereafter.

### 2.4 Data collection and propensity score matching

Baseline characteristics, including demographic variables, comorbid conditions, medication exposure, and laboratory data were ascertained within 5 years before the index date. Because depression risk in patients with hearing impairment may be influenced by underlying psychiatric, neurologic, sleep-related, and medical conditions, the propensity score model additionally included clinically relevant covariates, such as anxiety disorders, sleep disorders, chronic pain conditions, and other comorbidities potentially associated with both vitamin D status and subsequent depression risk (Supplementary Table 1). Diagnosis- and medication-based covariates were identified from available EHR records. Laboratory covariates were based on available measurements only, and missing laboratory values were not imputed. The analysis was therefore not a complete-case analysis requiring all patients to have complete laboratory data. Instead, threshold-defined laboratory covariates were constructed among patients with recorded measurements, and the availability of major laboratory variables are reported in Supplementary Table 2. Measured BMI ≥30 kg/m^2^ and ICD-10-CM-coded overweight or obesity were treated as distinct covariates; the former was derived from available BMI measurements, whereas the latter reflected diagnosis-coded obesity or overweight status. Propensity score matching was conducted in a 1:1 manner using a greedy nearest-neighbor algorithm with a caliper width of 0.1 standard deviations of the logit of the propensity score. Missing data were not imputed. Patients without a recorded value for a given laboratory test were classified as not meeting the prespecified laboratory threshold; thus, laboratory missingness was handled within the threshold-based binary covariate structure used for propensity score modeling rather than through explicit imputation. Balance in baseline covariates after matching was assessed by standardized mean differences (SMDs), and an SMD below 0.1 was regarded as acceptable.

### 2.5 Primary and secondary outcomes

The primary outcome was incident overall depression, defined by the first occurrence of diagnostic codes for a depressive episode (ICD-10-CM: F32) or recurrent major depressive disorder (F33). Secondary outcomes included depressive episode (F32) and recurrent major depressive disorder (F33) analyzed separately, all-cause mortality, and a composite outcome of suicidal behavior or ideation (R45.851), suicide attempt (T14.91), or intentional self-harm (X71–X83).

Given the established link between VDD and bone fragility, osteoporotic fractures (M80) were included as a positive control outcome. Acute appendicitis (K35), which has no known biological association with vitamin D status, was used as a negative control outcome to evaluate potential residual confounding. Healthcare utilization was evaluated as a time-to-event validation outcome, defined as the first documented healthcare encounter during follow-up. Encounter records available in TriNetX may include outpatient visits, inpatient encounters, and emergency visits captured by participating healthcare organizations; however, this validation analysis was not stratified by encounter type or psychiatric consultation. This analysis was included to assess whether differential clinical surveillance could have contributed to outcome detection. Because this outcome was analyzed using Cox proportional hazards models, the reported estimate reflects the relative hazard of having a first recorded healthcare encounter during follow-up, rather than the total number or rate of healthcare visits. To support exposure validity, subsequent clinical diagnoses of VDD after cohort entry were evaluated as an exposure validation outcome. Outcomes were assessed from 1 to 12 years after the index date to ensure temporal separation between exposure and outcome. Patients were censored at the time of death or at the end of the observation period, whichever occurred first.

### 2.6 Sensitivity, subgroup, and exposure-gradient analyses

Several prespecified sensitivity analyses were performed to examine the robustness of the primary association under alternative analytical conditions. First, Model I restricted the study period to 2016–2023. This analysis was intended to reduce potential heterogeneity arising from changes in laboratory practice, coding behavior, and clinical management over the longer study window and to assess whether the observed association remained consistent in a more contemporary cohort. Second, Model II was limited to patients with VDD diagnosed in medical centers. The rationale for this analysis was to improve diagnostic consistency and data completeness, as medical centers may provide more standardized laboratory testing, diagnostic coding, and follow-up than other healthcare settings. Third, Model III restricted the analysis to patients with conductive hearing impairment. This analysis was conducted to evaluate whether the main findings were reproducible within a more specific hearing-impairment phenotype and were not driven by heterogeneity across different types of hearing loss.

Prespecified subgroup analyses were conducted according to sex (female vs. male), age group (18–65 vs. >65 years), and selected comorbidities, including hypertension, diabetes mellitus, obesity, sleep disorders, and chronic pain conditions, to explore potential effect modification. An exploratory exposure–gradient analysis was also performed by comparing patients with vitamin D insufficiency (20.0–29.9 ng/ml) with a reference group of patients with vitamin D sufficiency (≥30.0 ng/ml). This analysis was designed to assess whether depression risk increased progressively with lower vitamin D levels. All sensitivity, subgroup, and exposure-gradient analyses were considered exploratory and were interpreted as supportive analyses rather than confirmatory tests.

### 2.7 Supplementary first-test-based analysis

To address the potential influence of cohort-entry definition, we performed a supplementary analysis in which cohort classification was based on the first recorded 25(OH)D test rather than the first qualifying 25(OH)D result. Patients whose first recorded 25(OH)D value met the VDD threshold were assigned to the VDD cohort, whereas patients whose first recorded value met the vitamin D sufficiency threshold were assigned to the control cohort. Follow-up and outcome definitions were otherwise consistent with the primary analysis, including the 1-year landmark period and assessment of depression-related outcomes, mortality, positive and negative control outcomes, healthcare utilization, and subsequent VDD diagnosis.

### 2.8 Statistical analysis

Associations between vitamin D status and study outcomes were estimated using Cox proportional hazards models and are presented as hazard ratios (HRs) with 95% confidence intervals (CIs). Cause-specific Cox models were used because the primary objective was to estimate etiologic associations between vitamin D status and incident depression. Death was treated as a censoring event in the time-to-event analyses. Analyses were based on available data, and no imputation was applied for missing values. The proportional hazards assumption was assessed using Schoenfeld residuals separately for the primary outcome and major secondary outcomes; a two-sided *P*-value >0.05 was considered to indicate no substantial violation of the assumption. Cumulative event-free survival was estimated using the Kaplan–Meier method, and between-group differences were compared using the log-rank test. The primary effect estimate was derived from the propensity score–matched cohort. Separately, an exploratory multivariable Cox regression model was fitted in the unmatched eligible cohort to assess whether VDD was associated with incident depression after covariate adjustment in the full analytic population.

To evaluate the potential influence of unmeasured confounding, *E*-values were calculated for the primary outcome. The *E*-value represents the minimum strength of association that an unmeasured confounder would need to have with both the exposure and outcome, above and beyond the measured covariates, to fully explain the observed association. For the primary endpoint, results were considered statistically significant if the two-sided *P*-value was below 0.05. Analyses of secondary outcomes, control outcomes, sensitivity models, and subgroup comparisons were considered exploratory; therefore, no formal adjustment for multiple comparisons was performed.

## 3 Results

### 3.1 Patient selection and baseline characteristics

**Figure 1:**
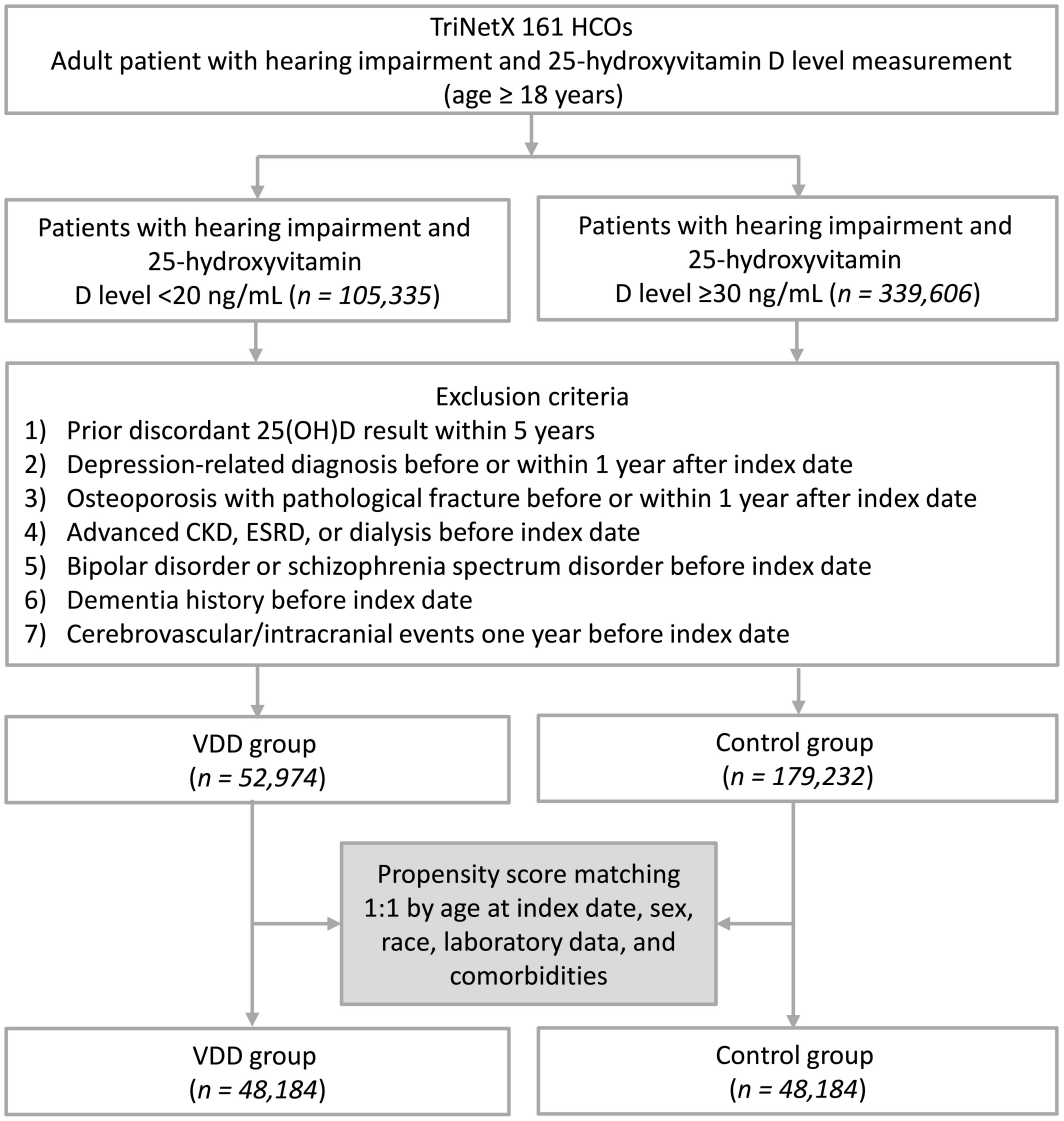
Flowchart of patient selection. HCOs, healthcare organizations; VDD, vitamin D deficiency; CKD, chronic kidney disease; ESRD, end-stage renal disease.

After applying the exclusion criteria, a total of 52,974 patients with VDD and 179,232 controls with vitamin D sufficiency met the initial eligibility criteria (Figure 1). Following 1:1 propensity score matching, 48,184 patients remained in each cohort. Before matching, several baseline characteristics differed substantially between the two groups, including age at index (53.7 ± 22.6 vs. 64.5 ± 17.0 years; *SMD* = 0.540), proportion identifying as White (58.1% vs. 80.4%; *SMD* = 0.498), prevalence of overweight and obesity (26.2% vs. 16.3%; *SMD* = 0.243), disorders of the thyroid gland (17.0% vs. 23.7%; *SMD* = 0.167), and nicotine dependence (9.3% vs. 5.1%; *SMD* = 0.163). These pre-matching differences indicate substantial baseline imbalance and underscore the potential for confounding by demographic and clinical factors. After propensity score matching, all measured covariates achieved acceptable balance, with all SMDs falling below 0.1. The matched cohorts were comparable in terms of demographic characteristics, comorbidity burden, laboratory parameters, and medication exposure (Table 1).

**Table 1:** Characteristics of patients with vitamin D deficiency and controls.

Variables	Before matching			After matching		
	VDD group (*n* = 52,974)	Control group (*n* = 179,232)	SMD^†^	VDD group (*n* = 48,184)	Control group (*n* = 48,184)	SMD^†^
Patient characteristics						
Age at index (years)	53.7 ± 22.6	64.5 ± 17.0	0.540	56.1 ± 21.5	56.0 ± 21.3	0.001
BMI ≥30 kg/m^2^	19,533 (36.9)	48,393 (27.0)	0.213	17,276 (35.9)	17,445 (36.2)	0.007
Female	30,058 (56.7)	113,412 (63.3)	0.134	27,649 (57.4)	27,510 (57.1)	0.006
White	30,785 (58.1)	144,135 (80.4)	0.498	30,527 (63.4)	30,457 (63.2)	0.003
Black or African American	11,192 (21.1)	13,520 (7.5)	0.395	8,288 (17.2)	8,367 (17.4)	0.004
Asian	2,430 (4.6)	8,385 (4.7)	0.004	2,368 (4.9)	2,236 (4.6)	0.013
Encounter for general examination	18,172 (34.3)	74,121 (41.4)	0.146	17,248 (35.8)	17,395 (36.1)	0.006
Comorbidities/medication						
Essential (primary) hypertension	23,764 (44.9)	88,585 (49.4)	0.092	22,403 (46.5)	22,770 (47.3)	0.015
Dorsalgia	16,092 (30.4)	56,581 (31.6)	0.026	14,952 (31.0)	14,988 (31.1)	0.002
Overweight and obesity^‡^	13,876 (26.2)	29,262 (16.3)	0.243	11,611 (24.1)	11,590 (24.1)	0.001
Diabetes mellitus	10,582 (20.0)	30,560 (17.1)	0.075	9,680 (20.1)	9,807 (20.4)	0.007
Sleep disorders	10,376 (19.6)	34,117 (19.0)	0.014	9,445 (19.6)	9,443 (19.6)	0.000
Disorders of thyroid gland	9,014 (17.0)	42,478 (23.7)	0.167	8,700 (18.1)	8,824 (18.3)	0.007
Pain, not elsewhere classified	9,071 (17.1)	31,035 (17.3)	0.005	8,437 (17.5)	8,388 (17.4)	0.003
Anxiety disorders	8,444 (15.9)	28,690 (16.0)	0.002	7,804 (16.2)	7,908 (16.4)	0.006
Ischemic heart diseases	7,059 (13.3)	25,446 (14.2)	0.025	6,693 (13.9)	6,698 (13.9)	0.000
Tinnitus	5,962 (11.3)	19,884 (11.1)	0.005	5,626 (11.7)	5,644 (11.7)	0.001
Chronic kidney disease (CKD)	4,817 (9.1)	17,068 (9.5)	0.015	4,491 (9.3)	4,608 (9.6)	0.008
Nicotine dependence	4,938 (9.3)	9,177 (5.1)	0.163	4,175 (8.7)	4,211 (8.7)	0.003
Diseases of liver	4,273 (8.1)	11,979 (6.7)	0.053	3,909 (8.1)	3,878 (8.0)	0.002
Atrial fibrillation and flutter	3,679 (6.9)	13,932 (7.8)	0.032	3,501 (7.3)	3,498 (7.3)	0.000
Heart failure	3,665 (6.9)	9,600 (5.4)	0.065	3,326 (6.9)	3,351 (7.0)	0.002
COPD	3,523 (6.7)	10,040 (5.6)	0.044	3,239 (6.7)	3,255 (6.8)	0.001
Cerebrovascular diseases	3,462 (6.5)	12,152 (6.8)	0.010	3,231 (6.7)	3,216 (6.7)	0.001
COVID-19	1,813 (3.4)	5,870 (3.3)	0.008	1,690 (3.5)	1,688 (3.5)	0.000
Systemic connective tissue disorders	1,368 (2.6)	5,903 (3.3)	0.042	1,300 (2.7)	1,296 (2.7)	0.001
Alcohol related disorders	1,327 (2.5)	2,589 (1.4)	0.076	1,134 (2.4)	1,092 (2.3)	0.006
Malnutrition	1,276 (2.4)	2,595 (1.4)	0.070	1,114 (2.3)	1,160 (2.4)	0.006
Reduced mobility	537 (1.0)	1,444 (0.8)	0.022	475 (1.0)	508 (1.1)	0.007
Laboratory data^§^						
Hemoglobin ≥12 g/dl	36,139 (68.2)	122,554 (68.4)	0.003	33,338 (69.2)	33,581 (69.7)	0.011
Albumin ≤ 3.5 g/dl	9,320 (17.6)	22,231 (12.4)	0.146	8,173 (17.0)	8,218 (17.1)	0.002
HbA1c ≥9%	2,674 (5.0)	4,321 (2.4)	0.140	2,196 (4.6)	2,199 (4.6)	0.000
eGFR ≤ 60 ml/min/1.73 m^2^	9,861 (18.6)	34,481 (19.2)	0.016	9,223 (19.1)	9,398 (19.5)	0.009
C-reactive protein ≥10 mg/L	4,258 (8.0)	8,954 (5.0)	0.123	3,655 (7.6)	3,578 (7.4)	0.006
Medications						
Central nervous system medications	37,853 (71.5)	120,863 (67.4)	0.087	34,201 (71.0)	34,598 (71.8)	0.018
Cardiovascular medications	33,001 (62.3)	117,314 (65.5)	0.066	30,557 (63.4)	30,940 (64.2)	0.017
Benzodiazepine derivative sedatives/hypnotics	16,895 (31.9)	57,794 (32.2)	0.008	15,591 (32.4)	15,779 (32.7)	0.008
Vitamin D supplementation	7,564 (14.3)	38,746 (21.6)	0.192	7,274 (15.1)	7,757 (16.1)	0.028
Blood glucose lowering drugs, excl. insulins	7,098 (13.4)	20,065 (11.2)	0.067	6,469 (13.4)	6,532 (13.6)	0.004
Insulins and analogs	5,645 (10.7)	12,662 (7.1)	0.127	4,925 (10.2)	4,915 (10.2)	0.001
Iron supplementation	4,153 (7.8)	10,006 (5.6)	0.090	3,574 (7.4)	3,625 (7.5)	0.004

Data are presented as *n* (%) or mean ± *SD*. SMD, standardized mean difference; BMI, body mass index; COPD, chronic obstructive pulmonary disease; eGFR, estimated glomerular filtration rate; HbA1c, hemoglobin A1c; VDD, vitamin D deficiency. †SMD values < 0.1 indicate adequate balance between groups.

^§^Laboratory covariates were based on available measurements only, without imputation. Thresholded values represent the proportion of the matched cohort with an available result meeting the specified threshold, rather than the prevalence in the entire population.

^‡^Measured BMI ≥30 kg/m^2^ and ICD-10-CM-coded overweight or obesity were treated as distinct covariates; the former was derived from available BMI measurements, whereas the latter reflected diagnosis-coded obesity or overweight status.

### 3.2 Outcomes

**Figure 2:**
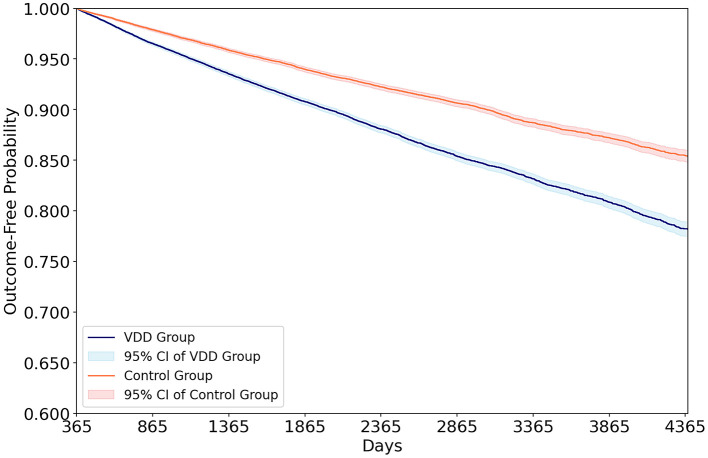
Kaplan–Meier curves for depression-free survival over 12 years. The VDD group demonstrated significantly lower depression-free survival compared with the control group (log-rank *P* < 0.001). Shaded areas represent 95% confidence intervals. Follow-up began 1 year after the index date (landmark period) and extended to 12 years. VDD, vitamin D deficiency; CI, confidence interval.

Over a follow-up period extending up to 12 years from the index date, incident overall depression occurred in 4,918 patients (10.21%) in the VDD group compared to 3,371 patients (7.00%) in the control group (Table 2). VDD was linked to a significantly higher risk of overall depression (*HR* 1.57; 95% *CI* 1.51–1.64; *P* < 0.001) (Figure 2). Consistent associations were observed for depressive episodes (*HR* 1.60; *P* < 0.001), recurrent depressive disorder (*HR* 1.62; *P* < 0.001), and the composite outcome of suicidal behavior, suicide attempt, or intentional self-harm (*HR* 1.47; *P* < 0.001). All-cause mortality was also higher in the VDD group (*HR* 1.40; *P* < 0.001).

**Table 2:** Association between vitamin D deficiency and 12-year depression risk.

Outcome	VDD group (*n* = 48,184)	Control group (*n* = 48,184)	*HR* (95% *CI*)	*P*-value	Absolute risk difference
	Events (%)	Events (%)			
Primary outcome					
Overall depression	4,918 (10.21%)	3,371 (7.00%)	1.57 (1.51–1.64)	<0.001	+3.21%
Secondary outcomes					
Depression episode	4,449 (9.23%)	2,994 (6.21%)	1.60 (1.53–1.67)	<0.001	+3.02%
Recurrent depression	1,218 (2.53%)	792 (1.64%)	1.62 (1.48–1.77)	<0.001	+0.89%
Suicide/self-harm^†^	264 (0.55%)	188 (0.39%)	1.47 (1.22–1.77)	<0.001	+0.16%
Mortality	4,642 (9.63%)	3,484 (7.23%)	1.40 (1.34–1.46)	<0.001	+2.40%
Positive control outcomes					
Osteoporotic fracture	502 (1.04%)	340 (0.71%)	1.55 (1.35–1.78)	<0.001	+0.33%
Negative control outcomes					
Acute appendicitis	226 (0.47%)	212 (0.44%)	1.11 (0.92–1.34)	0.267	+0.03%
Healthcare utilization validation					
First healthcare encounter during follow-up^‡^	44,458 (92.27%)	45,698 (94.84%)	0.87 (0.86–0.88)	<0.001	−2.57%
Exposure validation					
Subsequent VDD diagnosis^§^	12,575 (26.10%)	1,458 (3.03%)	10.81 (10.23–11.41)	<0.001	+23.07%

Data are presented as *n* (%) for events and hazard ratios (HR) with 95% confidence intervals (CI). VDD, vitamin D deficiency.

^†^Composite outcome of suicidal behavior, suicide attempt, and intentional self-harm.

^‡^Healthcare encounter was analyzed as a time-to-first-event outcome and reflects the first documented healthcare encounter during follow-up, rather than the total number or rate of visits.

^§^Subsequent VDD diagnosis was included as an exposure validation outcome.

For the control outcomes, osteoporotic fracture, prespecified as a positive control, was more common in the VDD group (*HR* 1.55; *P* < 0.001). In contrast, acute appendicitis, used as a negative control outcome, did not differ significantly between groups (*HR* 1.11; *P* = 0.267). The VDD group had a slightly lower hazard of a first documented healthcare encounter during follow-up (*HR* 0.87; 95% *CI*, 0.86–0.88), suggesting that the observed increase in depression risk was unlikely to be explained by greater healthcare surveillance in the VDD group. Furthermore, subsequent diagnoses of VDD were far more frequent in the VDD cohort (26.10% vs. 3.03%; *HR* 10.81), further supporting the stability and validity of the exposure classification.

The *E*-value for the primary outcome was 2.52 (lower confidence interval limit, 2.39), indicating that an unmeasured confounder would need to be associated with both VDD and depression by a HR of at least 2.52-fold each to fully explain the observed association.

### 3.3 Sensitivity analyses

The association between VDD and incident depression remained robust across all three sensitivity models (Table 3). In Model I, which restricted the study period to 2016–2023 (*n* = 39,620 per group), the HR for overall depression was 1.55 (*P* < 0.001). In Model II, which was limited to individuals diagnosed with VDD in medical centers (*n* = 37,966 per group), the HR was 1.63 (*P* < 0.001). In Model III, which was restricted to individuals with conductive hearing impairment (*n* = 26,740 per group), the HR was 1.65 (*P* < 0.001). Associations with depression subtypes, suicide/self-harm, and mortality were directionally consistent across all models.

**Table 3:** Sensitivity analyses of the association between vitamin D deficiency and 12-year depression risk.

Outcomes	Model I		Model II		Model III	
	*HR* (95% *CI*)	*P*-value	*HR* (95% *CI*)	*P*-value	*HR* (95% *CI*)	*P*-value
Overall depression	1.55 (1.48–1.63)	<0.001	1.63 (1.55–1.71)	<0.001	1.65 (1.56–1.75)	<0.001
Depression episode	1.56 (1.48–1.65)	<0.001	1.63 (1.55–1.72)	<0.001	1.66 (1.56–1.77)	<0.001
Recurrent depression	1.71 (1.54–1.89)	<0.001	1.65 (1.49–1.84)	<0.001	1.77 (1.57–2.00)	<0.001
Suicide/self-harm^†^	1.47 (1.17–1.83)	0.001	1.55 (1.25–1.91)	<0.001	1.61 (1.25–2.08)	<0.001
Mortality	1.41 (1.33–1.49)	<0.001	1.36 (1.30–1.43)	<0.001	1.33 (1.25–1.41)	<0.001

Data are presented as hazard ratios (HR) with 95% confidence intervals (CI). Model I: study period restricted to 2016–2023 (*n* = 39,620 per group). Model II: restricted to patients diagnosed with vitamin D deficiency in medical centers (*n* = 37,966 per group). Model III: restricted to patients with conductive hearing impairment (*n* = 26,740 per group).

^†^Composite outcome of suicidal behavior, suicide attempt, and intentional self-harm.

### 3.4 Subgroup analyses

**Figure 3:**
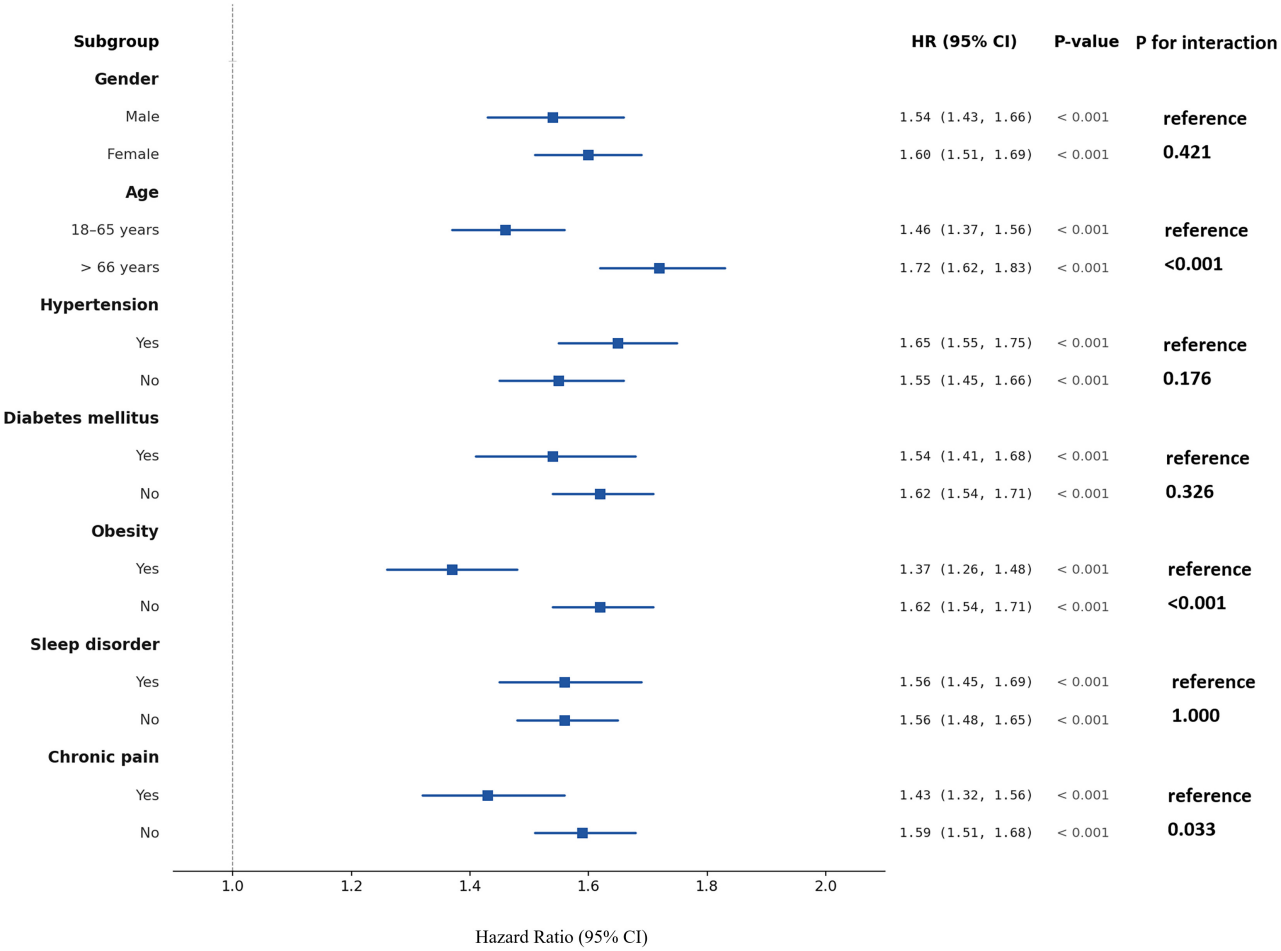
Forest plot of subgroup analyses for the association between vitamin D deficiency and incident depression. Hazard ratios (HRs) with 95% confidence intervals (CIs) are shown for each subgroup. *P* for interaction values were calculated to assess effect modification across subgroups. Subgroups were defined by sex (male vs. female), age (18–65 vs. >65 years), hypertension, diabetes mellitus, obesity, sleep disorders, and chronic pain conditions.

The association between VDD and incident depression was broadly consistent across most of the examined subgroups (Figure 3). All subgroup-specific hazard ratios remained statistically significant (all *P* < 0.001). No significant interactions were observed for sex (*P* for interaction = 0.421), hypertension (*P* = 0.176), diabetes mellitus (*P* = 0.326), or sleep disorders (*P* = 1.000). However, significant effect modification was identified for age (*P* for interaction < 0.001), obesity (*P* < 0.001), and chronic pain (*P* = 0.033). The association was stronger among patients aged >65 years (*HR* 1.72) than those aged 18–65 years (*HR* 1.46), more pronounced among patients without obesity (*HR* 1.62) than those with obesity (*HR* 1.37), and numerically larger in patients without chronic pain (*HR* 1.59) compared with those with chronic pain (*HR* 1.43). Despite these differences in magnitude, the association persisted across all subgroups.

### 3.5 Adjusted hazard ratios for depression from multivariable cox regression

In an exploratory multivariable Cox regression analysis conducted in the unmatched eligible cohort, VDD remained associated with incident depression after covariate adjustment (*HR*, 1.67; *P* < 0.001; Table 4) Pain not elsewhere classified (*HR* 1.59; *P* < 0.001) and sleep disorders (*HR* 1.42; *P* < 0.001) were also strong predictors. Male sex was associated with a lower risk of depression (*HR* 0.81; *P* < 0.001), whereas increasing age was associated with a modestly reduced risk (*HR* 0.99; *P* < 0.001). Other covariates with statistically significant but modest associations included essential hypertension (*HR* 1.18), other anemias (*HR* 1.16), malnutrition (*HR* 1.16), diabetes mellitus (*HR* 1.13), being overweight or obese (*HR* 1.10), and disorders of the thyroid gland (*HR* 1.04). Chronic kidney disease and liver disease were not significantly associated with depression risk.

**Table 4:** Adjusted hazard ratios for depression from multivariable Cox regression model.

Variable	*HR* (95% *CI*)	*P*-value
VDD vs. control groups	1.67 (1.61–1.73)	<0.001
Male	0.81 (0.78–0.83)	<0.001
Age at index	0.99 (0.98–0.99)	<0.001
Anemias	1.16 (1.11–1.21)	<0.001
Essential (primary) hypertension	1.18 (1.14–1.22)	<0.001
Diabetes mellitus	1.13 (1.09–1.18)	<0.001
Overweight and obesity	1.10 (1.06–1.14)	<0.001
Sleep disorders	1.42 (1.37–1.48)	<0.001
Disorders of thyroid gland	1.04 (1.00–1.08)	0.043
Pain, not elsewhere classified	1.59 (1.53–1.65)	<0.001
Malnutrition	1.16 (1.02–1.31)	0.022
Chronic kidney disease (CKD)	1.04 (0.98–1.10)	0.173
Diseases of liver	1.04 (0.98–1.11)	0.154

Data are presented as hazard ratios (HRs) with 95% confidence intervals (CIs). The multivariable Cox regression model was fitted in the unmatched eligible cohort and included vitamin D deficiency status, demographic variables, and clinically relevant comorbidities. VDD, vitamin D deficiency; CKD, chronic kidney disease.

### 3.6 Exposure-gradient analysis

In the exploratory exposure–gradient analysis comparing patients with vitamin D insufficiency [25(OH)D 20.0–29.9 ng/ml; *n* = 70,067 per group] to those with vitamin D sufficiency (≥30.0 ng/ml), a graded pattern was observed (Table 5). Vitamin D insufficiency was associated with a modestly elevated risk of overall depression (*HR* 1.37; *P* < 0.001), depressive episode (*HR* 1.40; *P* < 0.001), and recurrent depressive disorder (*HR* 1.34; *P* < 0.001). These effect estimates were numerically smaller than those observed in the primary analysis comparing VDD with vitamin D sufficiency, supporting an exposure-gradient pattern in which progressively lower vitamin D levels were associated with incrementally higher depression risk. The negative control outcome remained non-significant (*HR* 0.93; *P* = 0.418), whereas the positive control outcome retained its expected direction (*HR* 1.29; *P* < 0.001).

**Table 5:** Association between vitamin D insufficiency and depression risk during 12-year follow-up.

Outcome	VDI group (*n* = 70,067)	Control group (*n* = 70,067)	*HR* (95% *CI*)	*P*-value
	Events (%)	Events (%)		
Overall depression	6,610 (9.43)	4,962 (7.08)	1.37 (1.32–1.42)	<0.001
Depression episode	5,973 (8.53)	4,398 (6.28)	1.40 (1.34–1.45)	<0.001
Recurrent depression	1,581 (2.26)	1,192 (1.70)	1.34 (1.24–1.44)	<0.001
Suicide/self-harm^†^	337 (0.48)	249 (0.36)	1.36 (1.15–1.60)	<0.001
Mortality	5,979 (8.53)	5,149 (7.35)	1.17 (1.13–1.22)	<0.001
Osteoporotic fracture	769 (1.10)	600 (0.86)	1.29 (1.16–1.44)	<0.001
Acute appendicitis	263 (0.38)	283 (0.40)	0.93 (0.79–1.10)	0.418
First healthcare encounter during follow-up^‡^	65,836 (93.96)	66,506 (94.92)	0.92 (0.91–0.93)	<0.001
Subsequent VDD diagnosis^§^	8,747 (12.48)	2,842 (4.06)	3.30 (3.17–3.45)	<0.001

Data are presented as *n* (%) for events and hazard ratios (HR) with 95% confidence intervals (CI). VDI, vitamin D insufficiency, defined as serum 25-hydroxyvitamin D level of 20.0–29.9 ng/ml. The control group comprised patients with vitamin D sufficiency (≥30.0 ng/ml).

^†^Composite outcome of suicidal behavior, suicide attempt, and intentional self-harm.

^‡^Healthcare encounter was analyzed as a time-to-first-event outcome and reflects the first documented healthcare encounter during follow-up, rather than the total number or rate of visits.

^§^Subsequent VDD diagnosis was included as an exposure validation outcome.

### 3.7 Supplementary first-test-based analysis

In the supplementary first-test-based analysis (Supplementary Table 3), 38,353 patients were included in each matched cohort. VDD remained associated with a higher risk of overall depression, although the effect estimate was attenuated compared with the primary analysis (*HR*, 1.18; 95% *CI*, 1.14–1.23; *P* < 0.001). Similar associations were observed for depressive episode (*HR*, 1.19; *P* < 0.001), recurrent depression (*HR*, 1.25; *P* < 0.001), and mortality (*HR*, 1.25; *P* < 0.001), whereas suicide/self-harm was not statistically significant (*HR*, 1.07; *P* = 0.419).

For validation outcomes, osteoporotic fracture, the positive control outcome, was unexpectedly lower in the VDD group (*HR*, 0.83; *P* = 0.003), while acute appendicitis, the negative control outcome, was significantly higher (*HR*, 1.29; *P* = 0.026). First healthcare encounter during follow-up was less frequent in the VDD group (*HR*, 0.90; *P* < 0.001), whereas subsequent VDD diagnosis remained substantially more frequent (*HR*, 6.04; *P* < 0.001). These findings suggest that the association between VDD and depression persisted under first-test-based classification, but the attenuated effect estimates and inconsistent control-outcome pattern suggest sensitivity to cohort-entry definition and possible exposure misclassification.

## 4 Discussion

In this large-scale retrospective cohort study using the TriNetX Global Collaborative Network, we found that VDD was significantly associated with an increased risk of incident depression over a 12-year follow-up period among adults with hearing impairment. This association remained robust across multiple sensitivity analyses, was consistent in a multivariate Cox regression model (supplemental analysis), and was supported by an exposure-gradient pattern in which vitamin D insufficiency conferred a smaller but still significant elevation in risk compared to VDD. To the best of our knowledge, this is the first study to specifically examine the association between VDD and long-term depression risk in patients with pre-existing hearing impairment.

Our finding that VDD was associated with a 57% higher risk of overall depression (*HR* 1.57) is broadly consistent with prior evidence from general adult populations. For example, a meta-analysis of cohort studies (19) reported a pooled HR of 2.21 for depression in the lowest vs. highest vitamin D categories; however, a direct comparison is limited by differences in study populations, vitamin D thresholds, and analytic methods. In absolute terms, overall depression occurred in 10.21% of patients in the VDD group and 7.00% of controls, corresponding to an absolute risk difference of 3.21 percentage points over 12 years, or approximately 32 additional depression events per 1,000 patients. Our results extend this literature by showing that the association also persists in adults with hearing impairment, a population already vulnerable to depression. However, statistical significance should not be equated directly with clinical significance; the clinical importance of these findings depends on the absolute risk increase, baseline vulnerability of the population, feasibility of vitamin D assessment, and whether correction of VDD can reduce depression risk in prospective interventional studies.

Beyond its association with overall depression, VDD was also associated with an elevated risk of suicidal behavior, suicide attempts, or intentional self-harm (*HR* 1.47); this finding remained robust across all sensitivity analyses. Nevertheless, the absolute event rates were low, corresponding to an absolute risk difference of only 0.16% points. Therefore, this result should be interpreted as a signal requiring cautious contextualization rather than evidence of a large absolute increase in suicidal behavior. Prior evidence on VDD and suicidal outcomes has been inconclusive. A recent systematic review and meta-analysis (20) found that VDD was not significantly associated with the risk of suicide, suicide attempts, or suicidal ideation, although individuals with suicidal behavior had significantly lower vitamin D levels than controls. In contrast, a large retrospective cohort study of US veterans showed that vitamin D supplementation was associated with a substantially lower risk of suicide attempts and intentional self-harm, particularly among those with low baseline vitamin D levels (21). Our findings add to the literature by suggesting that, in adults with hearing impairment, the association between VDD and severe depression-related outcomes may be more readily detectable in a population with heightened psychosocial vulnerability.

Several biological mechanisms may underlie the observed association. Vitamin D receptors and the activating enzyme 1α-hydroxylase (CYP27B1), which converts 25(OH)D to its active form, are expressed in multiple brain regions relevant to mood regulation, including the prefrontal cortex and hippocampus; vitamin D signaling has also been implicated in limbic and stress-responsive circuits such as the amygdala (22, 23). Vitamin D has been proposed to modulate serotonergic neurotransmission, at least in part through transcriptional activation of tryptophan hydroxylase 2 (TPH2), the rate-limiting enzyme for serotonin synthesis in the brain; accordingly, deficiency may contribute to impaired central serotonergic signaling (24–26). Additionally, vitamin D has anti-inflammatory and antioxidant effects, whereas its deficiency has been linked to elevated interleukin-6 and tumor necrosis factor-α, two cytokines implicated in the pathophysiology of depression (27–29). In the context of hearing impairment, sensory deprivation may further contribute to depression vulnerability through reduced auditory input, altered corticolimbic processing, increased cognitive load, and social disconnection, which may interact with neuroinflammatory and neurotransmitter-related pathways. Beyond these neuropsychiatric pathways, vitamin D may be relevant to auditory health in this study population. Low vitamin D levels have been associated with sudden sensorineural hearing loss, poorer hearing-related outcomes, and increased tinnitus risk (30, 31), suggesting that VDD may contribute to hearing-related symptom burden as well as mood vulnerability. These factors may be compounded by communication difficulties, social withdrawal, and functional decline, potentially amplifying depression risk.

Subgroup analyses revealed notable effect modification by age, obesity, and chronic pain status. The stronger association observed in patients aged >65 years may reflect, in part, age-related reductions in cutaneous vitamin D synthesis, reduced outdoor activity (32), and the cumulative psychosocial burden of progressive hearing loss in older adults. The weaker association observed among patients with obesity should be interpreted cautiously. Although vitamin D sequestration in adipose tissue and reduced bioavailability may be possible explanations (33), this interaction may also reflect residual confounding, differences in clinical monitoring, or other unmeasured metabolic factors. The weaker association observed among patients with chronic pain may suggest that in this subgroup, pain-related disability and analgesic medication use represent dominant contributors to depression risk, partially overshadowing the contribution of vitamin D status. Importantly, all subgroup analyses showed a statistically significant association in the same direction, supporting the consistency of the findings across the examined strata.

Exposure–gradient analysis provided further support for the robustness of our findings. Vitamin D insufficiency was associated with a 37% increased risk of depression compared to a 57% increased risk in the VDD group. This graded pattern is consistent with a dose–response relationship and strengthens the biological and epidemiologic plausibility of the association. Moreover, acute appendicitis, used as a negative control outcome, showed no significant between-group difference in either the primary or gradient analyses, arguing against residual confounding or systematic bias as a complete explanation for the results. In contrast, osteoporotic fractures, used as a positive control outcome, demonstrated the expected association with VDD, supporting the validity of the exposure definition. The VDD group also had a lower hazard of a first documented healthcare encounter during follow-up. Although this finding does not exclude residual detection bias, it suggests that the higher observed depression risk is unlikely to be fully explained by greater clinical surveillance in the VDD group.

This study has several methodological strengths, including a large propensity score–matched cohort, extended follow-up, and a 1-year landmark design to improve temporal separation between vitamin D assessment and depression ascertainment. The use of positive and negative control outcomes, healthcare utilization assessment, and E-value analysis further supported the robustness of the findings, although the results remain subject to the inherent limitations of observational electronic health record-based research.

Several limitations should be acknowledged. First, as an observational study, the findings demonstrate an association and cannot establish a causal relationship between VDD and depression. The substantial pre-matching imbalance in age, race, and other baseline characteristics indicates that the VDD and sufficiency cohorts differed meaningfully before adjustment. Although propensity score matching improved measured covariate balance, residual confounding may persist because socioeconomic status, hearing aid use, hearing impairment severity, sunlight exposure, physical activity, social isolation, and nutritional quality were not consistently available in TriNetX. These unmeasured variables are particularly important in the present population because reduced outdoor activity, poorer diet or nutritional status, limited sunlight exposure, social withdrawal, and socioeconomic disadvantage may plausibly influence both vitamin D status and depression risk. Therefore, the observed association may partly reflect broader behavioral, functional, and social vulnerability rather than an independent effect of VDD itself. In addition, inclusion was restricted to patients who underwent serum 25(OH)D testing. This may introduce indication bias and healthcare-seeking bias, because testing may reflect clinical concern, comorbidity burden, access to care, or health-conscious behavior. Conditioning on vitamin D testing may also create collider bias if testing is influenced by factors related to both vitamin D status and depression risk. The lower hazard of a first documented healthcare encounter during follow-up does not eliminate the risk of healthcare-seeking bias, because all included patients had already interacted with the healthcare system sufficiently to undergo vitamin D testing. Thus, the findings may not be generalizable to all patients with hearing impairment.

Second, missing laboratory data were not imputed, and laboratory testing was not uniformly available for all patients. Although the analysis did not require complete laboratory data for inclusion, laboratory missingness may have been non-random and related to clinical indication, comorbidity burden, healthcare access, or healthcare-seeking behavior, which could introduce residual bias. In addition, vitamin D status was classified based on a single measurement, which may not capture longitudinal fluctuations. However, the substantially higher rate of subsequent VDD diagnoses in the VDD group (26.10% vs. 3.03%) supports the persistence of the exposure classification. Third, both exposure and outcome were ascertained using diagnostic codes and laboratory records within the TriNetX platform, which is subject to potential misclassification inherent to electronic health record-based studies. In addition, variability across participating healthcare organizations, including differences in laboratory calibration, vitamin D testing practices, diagnostic coding behavior, and follow-up patterns, may have introduced measurement heterogeneity.

Fourth, because the TriNetX network is composed primarily of healthcare organizations in the United States, the generalizability of our findings to other populations or healthcare settings may be limited. Fifth, although we adjusted for a comprehensive set of covariates, including anxiety disorders, sleep disorders, chronic pain, and medication use, residual confounding from unmeasured variables cannot be entirely excluded. In current study, antidepressant use was not used as an exclusion or matching criterion because these medications may be prescribed temporarily or for non-depression indications, and residual confounding related to antidepressant class, indication, dose, duration, adherence, or subclinical depressive symptoms cannot be fully excluded. Although the E-value analysis suggested that a moderately strong unmeasured confounder would be required to fully explain the primary association, it does not rule out residual confounding from strong or correlated unmeasured factors, such as socioeconomic status, physical activity, or frailty-related variables. In addition, subgroup analyses were exploratory and were not adjusted for multiple comparisons; therefore, significant interaction findings should be interpreted cautiously because false-positive findings are possible.

## 5 Conclusion

VDD was associated with a higher 12-year risk of incident depression among adults with hearing impairment, with a smaller association observed for vitamin D insufficiency. Because of the observational design, potential residual confounding, and single-measurement exposure classification, these findings should be interpreted as evidence of association rather than causality. From a clinical perspective, screening for VDD may help identify hearing-impaired patients who warrant closer mental health surveillance and broader risk assessment, but these observational findings do not establish that vitamin D supplementation prevents depression. Further prospective studies and randomized trials are needed to clarify whether vitamin D assessment or supplementation has clinical value for depression prevention in this population.

## Data Availability

The raw data supporting the conclusions of this article will be made available by the authors, without undue reservation.

## References

[1] Yan W Wang L Li C Meng Y Guo Q Li H. Bidirectional association between ADL disability and depressive symptoms among older adults: longitudinal evidence from CHARLS. Sci Rep. (2025) 15:7125. doi: 10.1038/s41598-025-91680-y 40021702 PMC11871208

[2] Rong J Wang X Cheng P Li D Zhao D. Global, regional and national burden of depressive disorders and attributable risk factors, from 1990 to 2021: results from the 2021 Global Burden of Disease study. Br J Psychiatry. (2025) 227:688–97. doi: 10.1192/bjp.2024.266 39809717

[3] Hohls JK König HH Quirke E Hajek A. Anxiety, depression and quality of life-a systematic review of evidence from longitudinal observational studies. Int J Environ Res Public Health. (2021) 18:12022. doi: 10.3390/ijerph182212022 34831779 PMC8621394

[4] Chan JKN Solmi M Lo HKY Chan MWY Choo LLT Lai ETH . All-cause and cause-specific mortality in people with depression: a large-scale systematic review and meta-analysis of relative risk and aggravating or attenuating factors, including antidepressant treatment. World Psychiatry. (2025) 24:404–21. doi: 10.1002/wps.21354 40948054 PMC12434377

[5] Shukla A Harper M Pedersen E Goman A Suen JJ Price C . Hearing loss, loneliness, and social isolation: a systematic review. Otolaryngol Head Neck Surg. (2020) 162:622–33. doi: 10.1177/0194599820910377 32151193 PMC8292986

[6] Henderson N Hodgson S Mulhern B Page K Sampson C. A qualitative systematic review of the impact of hearing on quality of life. Qual Life Res. (2025) 34:879–92. doi: 10.1007/s11136-024-03851-5 39579270 PMC11982117

[7] Reed NS Jiang K Deal JA. Hearing loss among older adults: epidemiology, disparities, and gaps in research. Annu Rev Public Health. (2026) 47:41–58. doi: 10.1146/annurev-publhealth-081524-110330 41160749 PMC12697576

[8] Tao Y Zhang H Wang D Li W. The prevalence and related factors of hearing loss among adults: a systematic review and meta-analyses. Ann Otol Rhinol Laryngol. (2025) 134:93–101. doi: 10.1177/00034894241293045 39707599

[9] Gong R Hu X Gong C Long M Han R Zhou L . Hearing loss prevalence and risk factors among older adults in China. Int J Audiol. (2018) 57:354–9. doi: 10.1080/14992027.2017.1423404 29400111

[10] Lawrence BJ Jayakody DMP Bennett RJ Eikelboom RH Gasson N Friedland PL. Hearing loss and depression in older adults: a systematic review and meta-analysis. Gerontologist. (2020) 60:e137–54. doi: 10.1093/geront/gnz009 30835787

[11] Wei J Li Y Gui X. Association of hearing loss and risk of depression: a systematic review and meta-analysis. Front Neurol. (2024) 15:1446262. doi: 10.3389/fneur.2024.1446262 39497727 PMC11532142

[12] Somoza-Moncada MM Turrubiates-Hernández FJ Muñoz-Valle JF Gutiérrez-Brito JA Díaz-Pérez SA Aguayo-Arelis A . Vitamin D in depression: a potential bioactive agent to reduce suicide and suicide attempt risk. Nutrients. (2023) 15:1765. doi: 10.3390/nu15071765 37049606 PMC10097210

[13] Menéndez SG Manucha W. Vitamin D as a modulator of neuroinflammation: implications for brain health. Curr Pharm Des. (2024) 30:323–32. doi: 10.2174/0113816128281314231219113942 38303529

[14] Akpinar S Karadag MG. Is vitamin D important in anxiety or depression? What is the truth? Curr Nutr Rep. (2022) 11:675–81. doi: 10.1007/s13668-022-00441-0 36097104 PMC9468237

[15] Dionisie V Gaman MA Anghele C Manea MC Puiu MG Stanescu S II . Vitamin D and depression in adults: a systematic review. Biomol Biomed. (2025) 25:2171–96. doi: 10.17305/bb.2025.12331 40322928 PMC12451993

[16] Li H Sun D Wang A Pan H Feng W Ng CH . Serum 25-hydroxyvitamin D levels and depression in older adults: a dose-response meta-analysis of prospective cohort studies. Am J Geriatr Psychiatry. (2019) 27:1192–202. doi: 10.1016/j.jagp.2019.05.022 31262683

[17] Ceolin G Moreira JD Quialheiro A Silva AAM d'Orsi E Rieger DK . Vitamin D serum concentration is prospectively associated with depressive symptoms in the EpiFloripa aging cohort study: a structural equation modeling approach. Braz J Psychiatry. (2024) 46:e20233153. doi: 10.47626/1516-4446-2023-3153 38251718 PMC11474441

[18] Assi S Twardzik E Deal JA Martin Ginis K Palta P Schrack JA . Hearing loss and physical activity among older adults in the United States. J Gerontol A Biol Sci Med Sci. (2024) 79:glad186. doi: 10.1093/gerona/glad186 37527509 PMC10733191

[19] Anglin RE Samaan Z Walter SD McDonald SD. Vitamin D deficiency and depression in adults: systematic review and meta-analysis. Br J Psychiatry. (2013) 202:100–7. doi: 10.1192/bjp.bp.111.106666 23377209

[20] Yu J Mohammad SN Khachatryan LG Mohammed JS Menon SV Kaur M . Risk of suicide, suicide attempt, and suicidal ideation among people with vitamin D deficiency: a systematic review and meta-analysis. BMC Psychiatry. (2025) 25:177. doi: 10.1186/s12888-025-06613-w 40000977 PMC11863558

[21] Lavigne JE Gibbons JB. The association between vitamin D serum levels, supplementation, and suicide attempts and intentional self-harm. PLoS ONE. (2023) 18:e0279166. doi: 10.1371/journal.pone.0279166 36724169 PMC9891532

[22] Mirarchi A Albi E Beccari T Arcuri C. Microglia and brain disorders: the role of vitamin D and its receptor. Int J Mol Sci. (2023) 24:11892. doi: 10.3390/ijms241511892 37569267 PMC10419106

[23] Cui X Eyles DW. Vitamin D and the central nervous system: causative and preventative mechanisms in brain disorders. Nutrients. (2022) 14:4353. doi: 10.3390/nu14204353 36297037 PMC9610817

[24] Huiberts LM Smolders K. Effects of vitamin D on mood and sleep in the healthy population: interpretations from the serotonergic pathway. Sleep Med Rev. (2021) 55:101379. doi: 10.1016/j.smrv.2020.101379 32987320

[25] Bostan ZZ Sare Bulut M Gezmen Karadag M. Can vitamin D reduce the need for SSRI by modulating serotonin synthesis?: a review of recent literature. Curr Nutr Rep. (2025) 14:39. doi: 10.1007/s13668-025-00630-7 40025236 PMC11872774

[26] Bonk S Hertel J Zacharias HU Terock J Janowitz D Homuth G . Vitamin D moderates the interaction between 5-HTTLPR and childhood abuse in depressive disorders. Sci Rep. (2020) 10:22394. doi: 10.1038/s41598-020-79388-7 33372187 PMC7769965

[27] Kouba BR Camargo A Gil-Mohapel J Rodrigues ALS. Molecular basis underlying the therapeutic potential of vitamin D for the treatment of depression and anxiety. Int J Mol Sci. (2022) 23:7077. doi: 10.3390/ijms23137077 35806075 PMC9266859

[28] Elgellaie A Thomas SJ Kaelle J Bartschi J Larkin T. Pro-inflammatory cytokines IL-1α, IL-6 and TNF-α in major depressive disorder: sex-specific associations with psychological symptoms. Eur J Neurosci. (2023) 57:1913–28. doi: 10.1111/ejn.15992 37070163

[29] Zhou J Li D Wang Y. Vitamin D deficiency participates in depression of patients with diabetic peripheral neuropathy by regulating the expression of pro-inflammatory cytokines. Neuropsychiatr Dis Treat. (2024) 20:389–97. doi: 10.2147/NDT.S442654 38436043 PMC10908276

[30] Sari E Tokat T Aliyeva A Karadag M Eren F Catli T . Assessment of vitamin D levels in patients with sudden sensorineural hearing loss. Ann Med Res. (2023) 30:538–44. doi: 10.5455/annalsmedres.2022.07.206

[31] Aliyeva A Han JS Kim Y Lim JH Seo JH Park SN. Vitamin D deficiency as a risk factor of tinnitus: an epidemiological study. Ann Otol Rhinol Laryngol. (2024) 133:647–53. doi: 10.1177/00034894241242330 38545900

[32] Chalcraft JR Cardinal LM Wechsler PJ Hollis BW Gerow KG Alexander BM . Vitamin D synthesis following a single bout of sun exposure in older and younger men and women. Nutrients. (2020) 12:2237. doi: 10.3390/nu12082237 32727044 PMC7468901

[33] Park CY Han SN. Vitamin D and obesity. Adv Food Nutr Res. (2024) 109:221–47. doi: 10.1016/bs.afnr.2023.12.006 38777414

