# Supplementary material for: Association of vitamin D deficiency with incident depression in patients with hearing impairment: an observational retrospective cohort study
Source: Front Nutr. 2026 Jun 10;13:1856953. doi: 10.3389/fnut.2026.1856953 (PMC13290609; doi:10.3389/fnut.2026.1856953)
Supplement: Supplementary file 1 [file Table_1.docx]

**Supplemental Table 1. Cohort Definition, Matching Variables, Outcomes, and Codes**

| Category | Variable | Codes / Definition |
| --- | --- | --- |
| Inclusion criteria | Age | ≥18 years |
|  | Hearing impairment | ICD-10: H90, H91 |
|  | Vitamin D measurement | TNX:9034, TNX:LG25965-1 |
|  | Study period | Jan 1, 2010 – Dec 31, 2023 |
| Exposure definition | Vitamin D deficiency | 25(OH)D <20 ng/mL (TNX:9034, TNX:LG25965-1) |
| Control definition | Normal vitamin D | 25(OH)D ≥30 ng/mL |
| Exclusion criteria | Prior depression | F32, F33 |
|  | Suicide / self-harm | R45.851, T14.91, X71–X83 |
|  | Osteoporosis fracture | M80 |
|  | End-stage kidney disease | N18.6 |
|  | CKD stage 4–5 | N18.4, N18.5 |
|  | Dialysis | Z99.2 |
|  | Bipolar disorder | F31 |
|  | Schizophrenia / psychotic disorders | F20–F29 |
|  | Dementia / Alzheimer disease | F01, F02, F03, G30 |
|  | Stroke / intracranial injury | I63, I61, S06 |
| Matching variables (PSM) | Age | Demographics |
|  | Sex | Demographics |
|  | Race | Demographics |
|  | Hypertension | I10 |
|  | Obesity | E66 |
|  | Diabetes mellitus | E08–E13 |
|  | Nicotine dependence | F17 |
|  | Ischemic heart disease | I20–I25 |
|  | Chronic kidney disease | N18 |
|  | Alcohol-related disorders | F10 |
|  | Cerebrovascular disease | I60–I69 |
|  | COPD | J44 |
|  | Malnutrition | E40–E46 |
|  | Sleep disorders | G47 |
|  | Heart failure | I50 |
|  | Atrial fibrillation | I48 |
|  | Anxiety disorders | F40–F48 |
|  | Liver disease | K70–K77 |
|  | General medical examination | Z00.0 |
|  | Reduced mobility | Z74.0 |
|  | Thyroid disorders | E00–E07 |
|  | Dorsalgia | M54 |
|  | Pain disorders | G89 |
|  | Tinnitus | H93.1 |
|  | Benzodiazepines | ATC CN302 |
|  | Insulin | ATC A10A |
|  | Oral hypoglycemics | ATC A10B |
|  | CNS medications | ATC CN000 |
|  | Cardiovascular medications | ATC CV000 |
|  | Vitamin D supplementation | ATC VT500 |
|  | Iron supplementation | ATC TN410 |
|  | Albumin | Lab 9045 |
|  | eGFR | LOINC 98979-8 |
|  | HbA1c | Lab 9037 |
|  | CRP | Lab 9063 |
|  | Hemoglobin | Lab 9014 |
|  | BMI | Lab 9083 |
| Primary outcome | Overall depression | F32, F33 |
| Secondary outcomes | Depression episode | F32 |
|  | Recurrent depression | F33 |
|  | Suicide | R45.851, T14.91, X71–X83 |
|  | Mortality | Deceased, R99 |
| Positive control outcome | Osteoporotic fracture | M80 |
| Negative control outcome | Appendicitis | K35 |
| Healthcare utilization | Medical visit | TNX:Visit |
| Follow-up definition | Outcome window | 1 to 12 years after index date |

**Supplemental Table 2. Laboratory measurement availability and threshold-defined laboratory covariates before and after propensity score matching**

| Laboratory variable / threshold | Before matching: VDD n (%) | Before matching: Control n (%) | SMD | After matching: VDD n (%) | After matching: Control n (%) | SMD |
| --- | --- | --- | --- | --- | --- | --- |
| Hemoglobin measured | 39,615 (74.78%) | 129,327 (72.16%) | 0.165 | 36,311 (75.36%) | 35,798 (74.29%) | 0.094 |
| Hemoglobin ≥ 12 g/dL | 36,139 (68.22%) | 122,554 (68.38%) | 0.003 | 33,338 (69.19%) | 33,581 (69.69%) | 0.011 |
| eGFR measured | 36,665 (69.21%) | 131,546 (73.39%) | 0.192 | 34,496 (71.59%) | 33,598 (69.73%) | 0.071 |
| eGFR≤ 60 mL/min/1.73 m² | 9,861 (18.62%) | 34,481 (19.24%) | 0.016 | 9,223 (19.14%) | 9,398 (19.50%) | 0.009 |
| BMI measured | 37,165 (70.16%) | 124,411 (69.41%) | 0.317 | 33,732 (70.01%) | 34,772 (72.17%) | 0.121 |
| BMI ≥30 kg/m^2^ | 19,533 (36.87%) | 48,393 (27.00%) | 0.213 | 17,276 (35.85%) | 17,445 (36.21%) | 0.007 |
| Albumin measured | 35,839 (67.65%) | 121,742 (67.92%) | 0.196 | 32,935 (68.35%) | 33,420 (69.36%) | 0.13 |
| Albumin≤ 3.5 g/dL | 9,320 (17.59%) | 22,231 (12.40%) | 0.146 | 8,173 (16.96%) | 8,218 (17.06%) | 0.002 |
| Hemoglobin A1c measured | 22,141 (41.80%) | 68,057 (37.97%) | 0.162 | 20,102 (41.72%) | 20,011 (41.53%) | 0.047 |
| Hemoglobin A1c≥ 9% | 2,674 (5.05%) | 4,321 (2.41%) | 0.14 | 2,196 (4.56%) | 2,199 (4.56%) | 0 |
| C-reactive protein measured | 8,370 (15.80%) | 22,957 (12.81%) | 0.187 | 7,506 (15.58%) | 7,209 (14.96%) | 0.037 |
| C-reactive protein≥ 10 mg/L | 4,258 (8.04%) | 8,954 (5.00%) | 0.123 | 3,655 (7.59%) | 3,578 (7.43%) | 0.006 |

**Supplemental Table 3. Association between vitamin D deficiency (VDD) and depression risk during 12-year follow-up using first-time VDD test**

| Outcome | VDI group  (n=38,353) | Control group  (n=38,353) | HR (95% CI) | P value |
| --- | --- | --- | --- | --- |
|  | Events (%) | Events (%) |  |  |
| Overall depression | 4,942 (12.89) | 4,219 (11.00) | 1.18 (1.14–1.23) | <0.001 |
| Depression episode | 4,479 (11.68) | 3,809 (9.93) | 1.19 (1.14–1.24) | <0.001 |
| Recurrent depression | 1,303 (3.40) | 1,035 (2.70) | 1.25 (1.15–1.36) | <0.001 |
| Suicide/self-harm† | 265 (0.69) | 244 (0.64) | 1.07 (0.90–1.28) | 0.419 |
| Mortality | 4,261 (11.11) | 3,385 (8.83) | 1.25 (1.20–1.31) | <0.001 |
| Osteoporotic fracture | 489 (1.28) | 578 (1.51) | 0.83 (0.74–0.94) | 0.003 |
| Acute appendicitis | 172 (0.45) | 132 (0.34) | 1.29 (1.03–1.62) | 0.026 |
| First healthcare encounter during follow-up‡ | 35,350 (92.17) | 36,172 (94.31) | 0.90 (0.88–0.91) | <0.001 |
| Subsequent VDD diagnosis§ | 9,044 (23.58) | 1,744 (4.55) | 6.04 (5.74–6.36) | <0.001 |

Data are presented as n (%) for events and hazard ratios (HR) with 95% confidence intervals (CI). VDD, vitamin D deficiency. †Composite outcome of suicidal behavior, suicide attempt, and intentional self-harm. ‡Healthcare encounter was analyzed as a time-to-first-event outcome and reflects the first documented healthcare encounter during follow-up, rather than the total number or rate of visits. §Subsequent VDD diagnosis was included as an exposure validation outcome.
